# Quality indicators for a geriatric emergency care (GeriQ-ED) – an evidence-based delphi consensus approach to improve the care of geriatric patients in the emergency department

**DOI:** 10.1186/s13049-020-00756-3

**Published:** 2020-07-16

**Authors:** Susanne Schuster, Katrin Singler, Stephen Lim, Mareen Machner, Klaus Döbler, Harald Dormann

**Affiliations:** 1 Faculty of Medicine, Friedrich-Alexander University Erlangen-Nürnberg, Erlangen, Germany; 2 Emergency Department, Klinikum Fürth, Fürth, Germany; 3 Institute for Nursing Research, Gerontology and Ethics, Lutheran University of Applied Sciences - Evangelische Hochschule Nürnberg, Nuremberg, Germany; 4 Institute for Biomedicine of Ageing, Friedrich-Alexander Universität Erlangen-Nürnberg, Nuremberg, Germany; 5 Geriatric Department - Medizinische Klinik 2, Geriatrie, Klinikum Nürnberg, Paracelsus Private Medical University, Nuremberg, Germany; 6 Academic Geriatric Medicine, University of Southampton, University Hospital Southampton NHS FT, Southampton, UK; 7 Charité – University of Medicine, Public Health Academy, Berlin, Germany; 8 Charité – University of Medicine, Lernzentrum, Medical Skills Lab, Berlin, Germany; 9 Competence Center Quality Management in Health Care, MDK Baden-Württemberg, Stuttgart, Germany

## Abstract

**Introduction:**

In emergency care, geriatric requirements and risks are often not taken sufficiently into account. In addition, there are neither evidence-based recommendations nor scientifically developed quality indicators (QI) for geriatric emergency care in German emergency departments. As part of the GeriQ-ED© research project, quality indicators for geriatric emergency medicine in Germany have been developed using the QUALIFY-instruments.

**Methods:**

Using a triangulation methodology, a) clinical experience-based quality aspects were identified and verified, b) research-based quality statements were formulated and assessed for relevance, and c) preliminary quality indicators were operationalized and evaluated in order to recommend a feasible set of final quality indicators.

**Results:**

Initially, 41 quality statements were identified and assessed as relevant. Sixty-seven QI (33 process, 29 structure and 5 outcome indicators) were extrapolated and operationalised. In order to facilitate implementation into daily practice, the following five quality statements were defined as the GeriQ-ED© TOP 5: screening for delirium, taking a full medications history including an assessment of the indications, education of geriatric knowledge and skills to emergency staff, screening for patients with geriatric needs, and identification of patients with risk of falls/ recurrent falls.

**Discussion:**

QIs are regarded as gold standard to measure, benchmark and improve emergency care. GeriQ-ED© QI focused on clinical experience- and research-based recommendations and describe for the first time a standard for geriatric emergency care in Germany. GeriQ-ED© TOP 5 should be implemented as a minimum standard in geriatric emergency care.

## Introduction

Every third patient admitted to prehospital emergency medicine and clinical emergency medicine is older than 65 years old [1–3]. Demographic changes have led to unique challenges faced by emergency care.

Functional decline, cognitive impairments, such as delirium or dementia, multiple comorbidities, frailty, falls and polypharmacy often result in negative health outcomes [4–8] It is known that in geriatric emergency patients, the risk of adverse outcomes such as hospital (re) admission, institutionalisation and mortality are increased compared to younger patients [9, 10].

The American College of Emergency Physicians (ACEP), the American Geriatrics Society (AGS), the Emergency Nurses Association (ENA) and the Society for Academic Emergency Medicine (SAEM) have developed guidelines for the care of older people in the emergency department (ED) [11]. However, in Australia and Europe, there are currently no consensus on which aspects of care to be included [7, 8, 12, 13]. To bring together both disciplines, geriatrics and emergency medicine, a European curriculum in geriatric emergency medicine was developed and approved by the European Union of Medical Specialists (UEMS) [14]. Additionally, a position paper by the German Society of Emergency Medicine (DGINA), the German Society of Geriatrics (DGG), the German Society of Gerontology and Geriatrics (DGGG), the Austrian Society of Geriatrics and Gerontology (ÖGGG) and the Swiss Society for Geriatrics (SFGG) have identified the need for further research and objective quality indicators (QIs) for geriatric emergency care [15]. A recent review highlighted that “a balanced, methodologically robust set of QIs for care of older persons in the ED” is needed [16]. Well-defined QIs will enable the assessment, benchmarking, and improvement of quality of care for geriatric emergency care patients [17].

During the development of the QIs, the following quality criteria were considered: scientific character, relevance and feasibility [18].

The aim of this paper is to describe the development process of QIs for the management of geriatric emergency patients and to provide a set of structure, process and outcome QIs (GeriQ-ED©).

## Methods

Triangulation methodology was applied for the development of the quality indicators, based on exploration of current evidence through a systematic literature search, and expert opinion from an interdisciplinary and interprofessional expert panel.

**Fig. 1:**
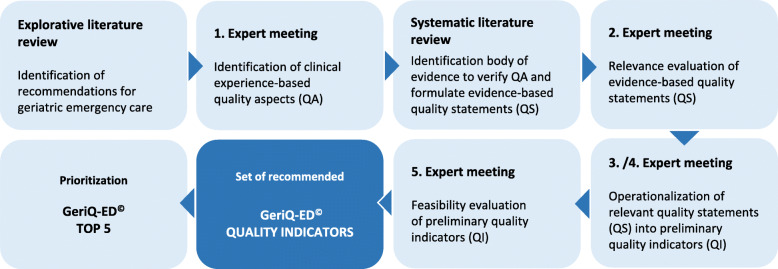
Methodical approach in the development of the GeriQ-ED© quality indicators

Action steps (Fig. 1):

- clinical experience-based quality aspects (QA) were identified and verified,
- evidence-based quality statements (QS) were formulated and assessed for relevance,
- preliminary quality indicators (QI) were operationalized and evaluated in order to recommend a feasible set of final quality indicators.

An exploratory literature review was conducted between 09/2014–10/2014 and an expert panel (*n* = 11) was established to contribute with its expertise on geriatric emergency care through a Delphi process [19]. The expert panel consisted of three emergency physicians and specially trained nurses, a geriatrician, a pharmacologist, a health economist and two participants who represented the views of older emergency patients.

At the first expert meeting (11/2014) a qualitative group discussion among the expert panel was conducted to identify relevant quality aspects of care for geriatric emergency patients. These quality aspects were evaluated using qualitative content analysis according to Mayring supported by MAXQDA [20]. A second systematic literature review (12/2014–03/2015) [search terms: `geriatric OR elderly OR senior` AND `emergency department´; databases: PubMed and CINAHL; inclusion criteria: published scientific papers, reviews, systematic reviews and meta-analyses between 2010 and 2015] was conducted to explore evidence for the potentially relevant quality aspects identified by the expert panel. Another aim of this systematic literature review was to verify the clinical experience-based quality aspects and to formulate evidence-based quality statements. During the second expert meeting (03/2015) an anonymized assessment of the relevance of all quality statements was conducted by the panel using a four-staged Likert-scale. The assessment took into consideration the importance, benefit and risk of each quality statement, based on the QUALIFY- instrument [19]. During the operationalisation process (third and fourth expert meeting - 05/2015 and 06/2015) preliminary quality indicators (structural, process or outcome indicators) including respective reference ranges were defined for every quality statement that was classified as relevant. To facilitate implementation of the preliminary quality indicators (QIs) into daily practice, QIs were assessed for their feasibility. To find a consensus during the fifth meeting (12/2015), experts used the anonymized two-step approach by RAND UCLA [21]. Finally, the panel was asked to define the QIs of five quality statements they regarded to be most important. These were prioritized as the “top five”.

## Results

The explorative literature review identified defined topics of geriatric emergency care [7, 8] QIs for selected areas in the field [13] and guidelines for geriatric emergency departments (ED) [11]. The potentially relevant quality aspects that were discussed during the first expert meeting were summarized into twelve different categories: education, staff, equipment, communication/information transfer, nursing care, medical treatment, geriatric screening, and risk factors such as falls, pain, cognitive impairment, medication and care needs (incontinence and the development of pressure sores).

The systematic literature review of potentially relevant quality aspects identified nine reviews, seven systematic reviews and two meta-analyses. Based on these results 41 quality statements were formulated. At the second meeting of the expert panel all 41 quality statements were assessed as being relevant. The following quality statements were rated as most relevant ($\overline{\mathrm{X}}$ = mean value):

- screening for delirium ($\overline{\mathrm{X}}$ 3,93)
- professional training requirements for emergency care staff ($\overline{\mathrm{X}}$ 3,90)
- barrier-free access to toilets with the possibility of supported transfer ($\overline{\mathrm{X}}$ 3,90)
- repetitive pain assessment including appropriate use of analgesics ($\overline{\mathrm{X}}$ 3,90)

During their third and fourth meeting the expert panel operationalized the 41 quality statements into 69 QIs. Apart from the statement ‘to implement a separate waiting area for geriatric patients’, the expert panel considered all other QIs as feasible at the fifth expert meeting.

Finally, a set of 67 clinical experience- and evidence-based GeriQ-ED© QIs (33 process QI, 29 structural QI and 5 outcome QI), which were relevant and feasible, were developed and operationalized (English translation of GeriQ-ED© available under additional online material). In 2017 GeriQ-ED© QIs have been published and are available for free on the website of the German Society of Emergency Medicine (DGINA) [22].

Table 1 shows an example of a GeriQ-ED© quality indicator regarding cognitive impairment/ delirium: 
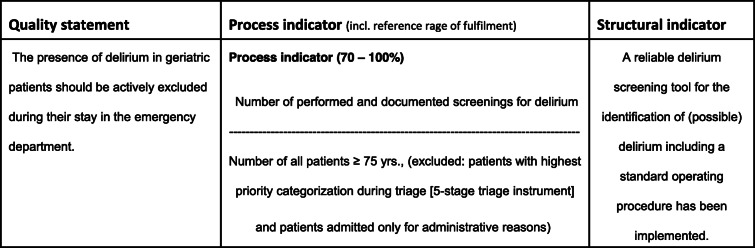

**Table 1:**
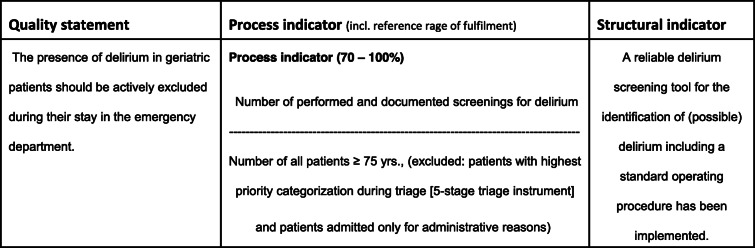
Example for GeriQ-ED©: cognitive impairment/delirium

In order to facilitate implementation into daily practice, the following five quality statements (associated with twelve quality indicators [22] https://www.dgina.de/news/geriq-c-quality-indicators-for-geriatric-emergency-care-entwicklung-von-qualitatsindikatoren-fur-die-versorgung-von-geriatrischen-notfallpatienten_63) were defined as the GeriQ-ED© TOP 5:

1. screening for delirium
2. taking a full medication history including an assessment of the indications
3. education of geriatric knowledge and skills to emergency staff
4. screening for patients with geriatric needs
5. identification of patients with risk of falls/ recurrent falls

### TOP 1: screening for delirium

Consequences of an undetected delirium include progressive deterioration of functional and cognitive impairment, and an increased risk of mortality [23, 24]. Studies show a strong association between the duration of delirium and mortality [25, 26]. Thus early detection of delirium in the emergency care setting is essential. Currently only a few screening-tools are validated and feasible in daily practice in the ED, such as the Confusion Assessment Method (CAM), the modified CAM-ED (mCAM-ED) [27, 28] and the 4-AT [29].

According to GeriQ-ED©, a standardized screening of delirium is recommended using a validated instrument that is feasible in the department settings. Although the exact timing of the screening in the emergency care process was not defined by the expert panel, delirium should be screened at the earliest time that is feasible in the ED management of the patient. In patients directly discharged from the ED, screening should be conducted prior to discharge. In addition, GeriQ-ED© recommends the implementation of a standardized management for patients at risk of delirium or patients with delirium including the documentation of risk factors as well as initial management of risk reduction as feasible in the ED [22] https://www.dgina.de/news/geriq-c-quality-indicators-for-geriatric-emergency-care-entwicklung-von-qualitatsindikatoren-fur-die-versorgung-von-geriatrischen-notfallpatienten_63.

### TOP 2: medication history including indications

Polypharmacy is common among older adults and is associated with an increased risk of adverse outcomes such as adverse drug reactions or medication errors. Adverse drug events (ADR) are a major cause of ED visits among older people [8, 30–32]. Nevertheless, most ADR are not detected. Studies have shown that up to 60% of all ADR are potentially avoidable [33]. Special attention should be given to the intake of anticoagulants, benzodiazepines, non-steroidal anti-inflammatory drugs, diuretics and antidepressants. These classes of drugs have in many cases been associated with complaints from older people who have been admitted to ED [32, 34–37].

Good clinical practice for the detection and prevention of ADRs in vulnerable patients include a detailed documentation and regular review of prescribed as well as over-the-counter medication by using a standardized medication reconciliation [38].

GeriQ-ED© recommends the implementation of a comprehensive medication management, including a detailed documentation of the current medication as well as a possible indication for each medication. Medication history and possible missing information on current medication should also be documented in the ED [22] https://www.dgina.de/news/geriq-c-quality-indicators-for-geriatric-emergency-care-entwicklung-von-qualitatsindikatoren-fur-die-versorgung-von-geriatrischen-notfallpatienten_63.

### TOP 3: staff education on geriatric knowledge and skills

Staff education level affects clinical outcomes in the emergency management [39]. In 2015 the Geriatric Section of the European Society for Emergency Medicine (EUSEM) together with the European Geriatric Medicine Society (EUGMS) established a joint task force to developed a curriculum for the care of older emergency patients (European Taskforce on Geriatric Emergency Medicine, ETFGEM). The aim was to outline relevant competencies in the care of older people, especially those with frailty. The curriculum incorporates knowledge on the physiology of ageing, common and atypical complaints, and the identification of geriatric syndromes or psychiatric needs of geriatric patients [14].

GeriQ-ED© confirms the need for an improvement in relevant competencies (knowledge and skills) of staff members who are involved in the care of older emergency patients and recommends for least 60% of the ED staff (physicians and nurses) the participation in at least one special geriatric training every year [22] https://www.dgina.de/news/geriq-c-quality-indicators-for-geriatric-emergency-care-entwicklung-von-qualitatsindikatoren-fur-die-versorgung-von-geriatrischen-notfallpatienten_63.

### TOP 4: screening for patients with geriatric needs

A recent meta-analysis showed that risk stratification of geriatric emergency patients is strongly limited by the lack of feasible and validated instruments. Existing instruments designed for risk stratification of older ED patients do not distinguish precisely between high- or low-risk groups [40]. However, as long as no better screening instruments are developed, it is recommended to use established and validated instruments [41].

GeriQ-ED© proposes the use one of the currently recommended evidence-based screening-tools in the ED to identify geriatric needs for action. Comprehensive geriatric assessment and extrapolated management have been shown to improve the outcome of older multimorbid people [42]. Further, GeriQ-ED© recommends a standardized implementation of management including screening of geriatric needs, and accurate documentation and information transfer. The timing to screen for geriatric needs was not defined [22] https://www.dgina.de/news/geriq-c-quality-indicators-for-geriatric-emergency-care-entwicklung-von-qualitatsindikatoren-fur-die-versorgung-von-geriatrischen-notfallpatienten_63.

### TOP 5: identification of patients with risk of falls/ recurrent falls

Appropriate evaluation of a fallen patient not only implies a thorough assessment for traumatic injuries, but also an assessment of potential causes and a stratification of future risk of falling [43, 44]. A proper assessment often requires a multidisciplinary team-approach. Currently no specific tools are recommended for the identification of potential risk factors [11]. The German Expert’s Standard for Fall and Fracture Prevention recommends an evaluation of person-, medication- and environmental-related risk factors such as fall history, the use of walking aids, depression, cognitive impairment and the long-term use of more than six different drugs [45].

GeriQ-ED© recommends the assessment and documentation of risk factors for falling during patient’s stay in the ED. The corresponding quality indicator recommends the documentation of > 80% of all patient cases in ED patients older than 70 years. Furthermore, it is recommended that every year more than 80% of the emergency nurses are trained on risk factors for falls [22] https://www.dgina.de/news/geriq-c-quality-indicators-for-geriatric-emergency-care-entwicklung-von-qualitatsindikatoren-fur-die-versorgung-von-geriatrischen-notfallpatienten_63.

## Discussion

High-quality geriatric emergency care is needed to ensure patient safety for this high-risk group. QIs are regarded as gold standard to measure, benchmark and improve emergency care. GeriQ-ED© focused on clinical experience and evidence-based recommendations and addressed the knowledge gap in this area. The proposed set of 67 GeriQ-ED©^−^QIs serves as a guidance for geriatric emergency care to ensure quality of care [7, 8, 46] and meets the recommendations made by the German position paper. For the first time QIs were developed that cover comprehensive geriatric emergency care and not only selected syndromes or fields of interest among geriatric emergency patients [13, 25, 47]. The operationalisation of quality statements into QIs enables an integration of them in existing documentation systems. The classification of quality aspects into twelve categories facilitates a thematic selection for special nursing or medical care issues.

In order to facilitate the implementation of QIs for older patient’s emergency care, the expert panel defined the top 5 out of the assigned 67 QIs.

### Implications for emergency care

GeriQ-ED© provide a set of 67 QIs including 33 process, 29 structure and 5 outcome indicators. They are intended as a framework for the provision of high quality geriatric emergency medicine adapted to the German emergency care. The QIs are intended to give the opportunity to assess own geriatric emergency care and to benchmark with other EDs. The QIs also give the opportunity to set individual goals for quality improvement in geriatric emergency care and to document the improvement accordingly.

To implement the 67 GeriQ-ED© QIs in the emergency care setting, further structural adaptations will be necessary. Individualised care of geriatric patients in order to improve the quality of care will require an adapted calculation of staff numbers in the EDs. Hospital management, leaders of EDs as well as ED nurse managers need to recognise that geriatric emergency patients ought to be considered as a highly vulnerable patient group with special needs that have to be addressed differently from usual care.

### Limitation

The process to develop the GeriQ-ED© QIs started in 2014. In 2017 the QIs were published in German [22]. Although GeriQ-ED© QIs refer to screening-tools based on current evidence (e.g. to screening for delirium or identification of geriatric needs) literature review for prior QIs had to be updated. In a recent systematic literature review (02/2020) no additional QIs were identified [search terms: `emergency care´ AND `geriatrics´; database: PubMed; inclusion criteria: published between 2015 and 2020].

The majority of the 67 GeriQ-ED© QIs are process- or structure indicators. The small number of outcome indicators was discussed with an expert for QI development. It was agreed that in the ED setting it is difficult to define outcome indicators due to the short stay of the patients and also the limited influence on the care received beyond the ED. Therefore, the development of outcome indicators in the field of emergency medicine is only possible with restrictions [12].

## Conclusions

Demographic changes imply big challenges for the emergency care. QIs for this special setting offer a solution to improve geriatric emergency care and patient’s safety. For the first time, GeriQ-ED© provides a comprehensive set of 67 QIs which addresses the specialist care needs of older people in the ED to improve patient care.

The methodical approach used for the development of GeriQ-ED© corresponds to required methodical quality criteria. They are evidence-based, relevant and feasible. GeriQ-ED© is based on a consensus among experts in the field. A prospective study is planned to evaluate the QIs in daily practice with a special focus on measuring criteria and feasibility.

However, in German Eds, GeriQ-ED© TOP 5 should be implemented as a minimum standard in geriatric emergency care.

## Supplementary information

**Additional file 1.**
