# Supplementary material for: Quality indicators for a geriatric emergency care (GeriQ-ED) – an evidence-based delphi consensus approach to improve the care of geriatric patients in the emergency department
Source: Scand J Trauma Resusc Emerg Med. 2020 Jul 16;28:68. doi: 10.1186/s13049-020-00756-3 (PMC7364502; doi:10.1186/s13049-020-00756-3)
Supplement: Supplementary file 1 — Additional file 1. [file 13049_2020_756_MOESM1_ESM.docx]

| **Quality categories** | **Quality statements** | **Process or outcome indicators**  (reference range) | **Structural indicators**  (Scoring: yes / no) |
| --- | --- | --- | --- |
| **1. Staffing**  **(and**  **2. Education)** | **1.1**  Emergency physicians should have basic knowledge in geriatrics. |  | **1.1 Structural indicator**  Documented evidence should be provided showing that annually, more than 60% of all ED physicians have attended educational meetings or received training in one of the following topics: |
|  |  |  | a) basic theoretical knowledge of geriatrics |
|  |  |  | b) soft skills related to dealing with geriatric patients/ relatives/ care givers or their respective relatives |
|  | **1.2**  Emergency nursing staff should have basic knowledge in geriatrics. |  | **1.2 Structural indicator**  Documented evidence should be provided showing that annually more than 60% of all ED nursing staff have attended educational meetings or received training in one of the following topics  a) basic theoretical knowledge of geriatrics  b) soft skills related to dealing with geriatric patients/ relatives/ care givers or their respective relatives |
|  | **1.3**  In the ED, collaboration with either geriatricians, geriatrics nurse specialists or a geriatric liaison service should be ensured. |  | **1.3 Structural indicator**  A framework for the involvement of geriatricians, allied health professional with specialist skills in geriatrics or a geriatric liaison service is available. |

|  | **1.4**  The involvement of voluntary or paid staff to provide support to geriatric patients who are awaiting medical review should be ensured. |  | **1.4 Structural indicator**  A service, which includes additional staffing for the care of geriatric patients during waiting times, is available. This additional care service will be provided during working days (Mon-Sat) at least 4hrs daily |
| --- | --- | --- | --- |
| **3. Equipment/ Supplies** | **3.1**  EDs should provide appropriate sitting and bedding facilities and therapy aids to support patient mobility. |  | **3.1 Structural indicator**  Appropriate sitting and bedding facilities, as well as an adequate supply of therapy aids to meet the needs of geriatric patients in the ED should be provided. |
|  | **3.2**  ED should be designed to be barrier-free and should facilitate a barrier free access to diagnostic facilities. |  | **3.2.1 Structural indicator**  All areas in the ED should be accessible for geriatric patients. |
|  |  |  | **3.2.2 Structural indicator**  Access from the ED to the diagnostic departments should be barrier-free. |
|  | **3.3**  Geriatric patients should be provided with accessible toilets, where supported transfer can be provided. |  | **3.3.1 Structural indicator**  The ED should have at least one accessible toilet. |
|  |  |  |  |
|  |  |  | **3.3.2 Structural indicator** |
|  |  |  | Equipment for supported transfer should be made available in at least one toilet within the ED. |

|  | **3.4**  ED should facilitate orientation for geriatric (cognitively impaired) patients, using signs and images (marking the bathroom etc.) |  | **3.4 Structural indicator**  In designated areas, provide signs and images to facilitate orientation for geriatric patients. |
| --- | --- | --- | --- |
|  | **3.5**  If there are no contraindications, geriatric patients should be given the opportunity to eat and drink with appropriate monitoring and with the necessary support during meal preparation (thickening of meals/ beverages, if necessary). |  | **3.5.1 Structural indicator**  Geriatric patients should be provided with snacks, drinking water and caloric beverages as required, if there is no clinical contraindication. |
|  |  |  | **3.5.2 Structural indicator** |
|  |  |  | A care standard will be provided, which ensures adequate observation of and assistance with oral intake (if necessary thickening of meals/beverages). |
|  | **3.6**  The interior design and colour scheme of the ED should take into consideration the needs of geriatric and cognitively impaired patients to help with orientation. |  | **3.6 Structural indicator**  The interior design and colour scheme of the ED should facilitate orientation for cognitively impaired patients. |

| **4. Medical Treatment** | **4.1**  Treatment of geriatric patients in the ED should be guided by current principles and clinical guidelines. Patients’ wishes or any advance directives should be clearly documented and confirmed that it is applicable to the current admission prior to initiating treatment. | **4.1.1 Process indicator (60 - 100%)**  Number of records on guideline-conforming therapies or justified deviations ----------------------------------------------------------------------------------------------------------------------  Number of patients ≥75 years old, excluding only administratively registered patients | **4.1.4 Structural indicator**  Availability of at least three SOPs (referring to guidelines-based treatment of geriatric patients), which have been drafted and approved by a geriatrician. |
| --- | --- | --- | --- |
|  |  | **4.1.2 Process indicator (70 - 100%)**  Number of patient or relative consultations with patient´s wishes clearly identified and documented  ----------------------------------------------------------------------------------------------------------------------  Number of patients ≥75 years old, excluding only administratively registered patients |  |
|  |  | **4.1.3 Process indicator (90 - 100%)**  Number of documented availability or non-availability of advance directives  ----------------------------------------------------------------------------------------------------------------------  Number of patients ≥75 years old, excluding only administratively registered patients |  |
|  | **4.2**  The contact number of the physician in charge of the patient should be clearly documented. | **4.2 Process indicator (90 - 100%)**  Number of documented physician contact during the ED stay  ----------------------------------------------------------------------------------------------------------------------  Number of patients ≥75 years old, excluding only administratively registered patients |  |
| **5. Nursing care** | **5.1**  During their stay in the ED, a dedicated named nurse should be assigned to oversee the care provision of geriatric patients to ensure continuity of care. | **5.1.1 Process indicator (90 - 100%)**  Number of documented continuous nursing contact during the ED stay  ----------------------------------------------------------------------------------------------------------------------  Number of patients ≥ 75 years old, excluding patients with the highest level of urgency (according to a 5 level triage tool) and only administratively registered patients | **5.1.2 Structural indicator**  Availability of a procedural instruction to ensure that each geriatric patient has a named nurse to oversee his or her care to ensure continuity of care. |

|  | **5.2**  Accompanying relatives or carers, if available, should be recorded and involved in the care process. | **5.2.1 Process indicator (90 - 100%)**  Number of records on accompanying and not accompanying relatives or carers ----------------------------------------------------------------------------------------------------------------------  Number of patients ≥75 years old, excluding only administratively registered patients | **5.2.2 Structural indicator**  A procedural instruction to involve relatives/carers in the care process of geriatric emergency patients is provided. This should occur:  a) at baseline  b) during treatment in the ED  (if stay lasts ≥ 2 hrs)  c) at the end of treatment process |
| --- | --- | --- | --- |
| **6. Communication / information** | **6.1**  The following information should be available in the ED (active retrieving may be necessary): Reason for presentation/ admission, current care status, cognitive status, extended directives (legal guardianship, advance directives, legal capacity), medical history/ care situation, current medication, contact details of relatives or care provider (for ED discharge). | **6.1.1 Process indicator (80 - 100%)**  Number of complete documentation of information (= eight items, 6.1.2.)  or justified missing documentation  ----------------------------------------------------------------------------------------------------------------------  Number of patients ≥ 75 years old, excluding patients with the highest level of urgency (according to a 5 level triage tool) and only administratively registered patients | **6.1.2 Structural indicator**  A complete documentation of the following information in the patient´s ED records:   1. reason for presentation/ admission 2. care status 3. cognitive status 4. extended directives (legal guardianship, advance directives, legal capacity) 5. medical history/ care needs 6. current medication 7. contact information of relatives 8. contact information of care providers (for ED discharge) |

|  | **6.2**  Throughout the ED stay, clear communication should be maintained with patients or their accompanying persons/carers. | **6.2.1 Outcome indicator (70 - 100%)**  Number of patients or accompanying persons/carers that are satisfied with the information provided during the ED stay  ----------------------------------------------------------------------------------------------------------------------  Number of all participants in the survey every two years | **6.2.4 Structural indicator**  A survey should be conducted once every two years to receive feedback from patients and their accompanying persons/carers about the information provided regarding their hospital stay. |
| --- | --- | --- | --- |
|  |  |  |  |
|  |  | **6.2.2 Outcome indicator (70 - 100%)** |  |
|  |  | Number of patients or accompanying persons/carers that are satisfied with the communication during the ED stay  ----------------------------------------------------------------------------------------------------------------------  Number of all participants in the survey every two years |  |
|  |  | **6.2.3 Outcome indicator (70 - 100%)** |  |
|  |  | Number of patients or accompanying persons/carers that are satisfied with communication upon discharge/ transfer  ----------------------------------------------------------------------------------------------------------------------  Number of all participants in the survey every two years |  |

|  | **6.3**  The following information should be forwarded at discharge/ transfer of the patient from the ED (to a geriatric specialist): Symptoms, diagnostic and treatment results, adjusted medication, and abnormalities / discrepancies during medication review, recommendation of follow-up treatment and contact telephone numbers. | **6.3.1 Process indicator (80 - 100 %)**  Number of transfer or discharge reports (= six factors) or justified missing documentation  ----------------------------------------------------------------------------------------------------------------------  Number of patients ≥ 75 years old, excluding patients with the highest level of urgency (according to a 5 level triage tool) and only administratively registered patients | **6.3.2 Structural indicator**  Availability of a transfer or discharge report which includes the following information:  a) summarized diagnostic, therapeutic and care process   1. with special consideration of geriatric aspects    - Screening and assessment results    - complete medication review, tested for plausibility, verified and if necessary adapted 2. completed documentation of information (from 6.3) 3. further recommendations (e.g. therapy recommendation, additional recommendation for further follow up-contacts |
| --- | --- | --- | --- |
|  |  |  |  |
|  |  |  |  |
| **7. Geriatric needs** | **7.1**  Geriatric patients should be screened using an evidence-based screening tool to identify specific geriatric needs. | **7.1.1 Process indicator (70 - 100%)**  Number of documented geriatric screenings  ----------------------------------------------------------------------------------------------------------------------  Number of patients ≥ 75 years old, excluding patients with the highest level of urgency (according to a 5 level triage tool) and only administratively registered patients | **7.1.2 Structural indicator**  An evidence-based geriatric screening tool is implemented and a standard for care provision is provided. |
|  | **7.2**  In patients who have been identified as having a specific geriatric need, a geriatric assessment during or promptly after the ED stay must be carried out by a healthcare professional trained specialist in geriatrics. | **7.2.1 Process indicator (70 - 100%)**  Number of recorded assessment results (=performed assessments) and recommendations for further assessment (=requested assessments)  ----------------------------------------------------------------------------------------------------------------------  Number of all patients with positive geriatric screening | **7.2.2 Structural indicator**  A procedural instruction is provided on how information transfer to geriatric specialists should be conducted in patients screened positive for geriatric needs. |

| **8. Risk factor: cognitive impairment** | **8.1**  During their stay in the ED, geriatric patients should be screened for delirium. | **8.1.1 Process indicator (70 - 100%)**  Number of documented delirium screenings  -----------------------------------------------------------------------------------------------------------------------  Number of patients ≥ 75 years old, excluding patients with the highest level of urgency (according to a 5 level triage tool) and only administratively registered patients | **8.1.2 Structural indicator**  A validated delirium-screening tool for the detection of delirium is implemented and a management plan for screening delirium is provided. |
| --- | --- | --- | --- |
|  | **8.2**  Identified precipitating factors causing delirium should be documented and if possible addressed. | **8.2.1 Process indicator (90 - 100%)**  Number of documented identified and unidentifiable causes for delirium  ----------------------------------------------------------------------------------------------------------------------  Number of patients ≥ 75 years with delirium |  |
|  |  | **8.2.2 Process indicator (60 - 100%)** |  |
|  |  | Number of documentation of treated/eliminated causes for delirium (for example treatment of infection, adaption of medication, sufficient hydration, pain treatment, bowel and urinary care)  ----------------------------------------------------------------------------------------------------------------------  Number of patients ≥ 75 years with delirium and minimal one documented cause for delirium |  |

|  | **8.3**  In cognitively impaired patients, aids for orientation as well as adapted support measures and communication should be readily available. |  | **8.3 Structural indicator**  A procedural instruction for adapted support measures and communication with cognitively impaired patients is provided and aligned to the following exemplary content:   1. adequate support for sensory impairment (spectacles, hearing aids) 2. orientation for patients (calendars, clocks, name tags) 3. clear, direct communication 4. support for continence care and mobilisation 5. adequate hydration/ nutrition 6. avoiding restraining patient at risk of self-harm or harming others 7. pharmacological treatment: : haloperidol rather than benzodiazepine, neuroleptic drugs as first line therapy 8. sensory stimulation (relaxation room, sufficient lighting, low noise levels, offer occupation) 9. if possible, avoid unnecessary invasive measures |
| --- | --- | --- | --- |

| **9. Risk factor polypharmacy** | **9.1**  In geriatric patients, a complete medication history including reference diagnoses and medications administered during the ED stay should be recorded and checked for indication, dosage and duration of therapy.  All information should be communicated in written form when the patient is transferred or discharged (if necessary missing information need to be gathered). | **9.1.1 Process indicator (90 - 100%)**  Number of documented medication history (documented medication or documentation that no medication is taken)  ----------------------------------------------------------------------------------------------------------------------  Number of patients ≥75 years old, excluding only administratively registered patients | **9.1.3 Structural indicator**  Possibility of recording a medication history including clear documentation of indications and diagnoses. |
| --- | --- | --- | --- |
|  |  | **9.1.2 Process indicator (90 - 100%)**  Number of medication review performed and documented references to diagnoses for any medication (including documentation, if no diagnosis reference is available)  ----------------------------------------------------------------------------------------------------------------------  Number of patients ≥ 75 years with documented medication | **9.1.4 Structural indicator**  A procedural instruction for conducting a complete medication history including a review of indication, dosage and duration of therapy is provided. |
|  | **9.2**  Changes or adaption in medication should be recorded and passed on in writing in case of transfer or discharge. | **9.2.1 Process indicator (90 - 100%)**  Number of documented medication changes / adaptions / administrations  with nomination of reason  ----------------------------------------------------------------------------------------------------------------------  Number of patients ≥ 75 years with documented medication changes / adaptions /  Administrations, excluding only administratively registered patients |  |
|  |  | **9.2.2 Process indicator (70 - 100%)** |  |
|  |  | Number of complete documented medication history passed on to follow-up care (including: complete medication history or documentation of gaps in medication history and documented change s/ adaptions in medication and administration of medication with rational)  ----------------------------------------------------------------------------------------------------------------------  Number of patients ≥ 75 years, excluding only administratively registered patients |  |

| **10. Risk factor falls** | **10.1**  During the ED stay geriatric patients should be screened for risk of falling. | **10.1.1 Process indicator (80 - 100%)**  Number of documented systematic assessments of potential fall risk factors based on current recommendations of the expert's standard “Fall prevention in nursing” (P1)  ----------------------------------------------------------------------------------------------------------------------  Number of patients ≥ 75 years, excluding patients with the highest degree of urgency (according to a 5 level triage instrument) and only administratively registered patients | **10.1.2 Structural indicator**  Appropriate evidence is provided that annually, more than 80% of ED nursing staff have knowledge on systematic data collection of fall risk factors. |
| --- | --- | --- | --- |
|  | **10.2**  In patients with an increased risk of falling, a falls-preventive environment should be ensured. | **10.2 Process indicator (90 - 100%)**  Number of documented adapted falls risk factors during the ED stay  (if not adapted a reason must be provided)  ----------------------------------------------------------------------------------------------------------------------  Number of patients ≥ 75 years with identified risk factors for falls |  |
|  | **10.3**  In geriatric patients a comprehensive physical examination with a special focus on recent or previous falls should be performed. | **10.3 Process indicator (80 - 100%)**  Number of documented physical examination with special focus on previous or recent falls  ----------------------------------------------------------------------------------------------------------------------  Number of patients ≥ 75 years, excluding patients with the highest degree of urgency (according to a 5 level triage instrument) and only administratively registered patients |  |
|  | **10.4**  In patients with a history of falls, an examination that considers differential diagnoses should be performed. | **10.4 Process indicator (70 - 100%)**  Number of documented examinations for differential diagnoses  ----------------------------------------------------------------------------------------------------------------------  Number of patients ≥ 75 years, with a previous fall, excluding patients with the highest degree of urgency (according to a 5 level triage instrument) and only administratively registered patients |  |

|  | **10.5**  Falls assessment should be recommended in current or previous falls when the patient is transferred or discharged. | **10.5 Process indicator (80 - 100%)**  Number of recorded falls assessment results (=performed assessments) and recommendations for further falls assessment (=requested assessments)  ----------------------------------------------------------------------------------------------------------------------  Number of patients ≥ 75 years with a fall or identified fall risk factors, excluding patients with the highest degree of urgency (according to a 5 level triage instrument) and only administratively registered patients |  |
| --- | --- | --- | --- |
| **11. Risk factor pain** | **11.1**  In all geriatric patients, including patients with cognitive impairment, assessment and management of pain should be performed and documented (incl. assessment of potentially adverse drug reactions of pain medication) | **11.1.1 Process indicator (80 - 100%)**  Number of documented pain assessments using an appropriate pain assessment instrument for patients with and without cognitive impairment  ----------------------------------------------------------------------------------------------------------------------  Number of patients ≥ 75 years, excluding patients with the highest degree of urgency (according to a 5 level triage instrument) and only administratively registered patients. | **11.1.4 Structural indicator**  Pain assessment tools suitable for geriatric patients with and without cognitive impairment in the ED are implemented and a standard for execution is present. |
|  |  | **11.1.2 Process indicator (90 - 100%)** | **11.1.5 Structural indicator** |
|  |  | Number of documented pain treatment according to ED specific guideline  for pain management in older people  ----------------------------------------------------------------------------------------------------------------------  Number of patients ≥ 75 years, with pain that calls for treatment | A pain management guideline specifically adapted to geriatric ED patients is available. |
|  |  | **11.1.3 Result indicator (70 - 100%)**  Number of documented diminished pain during repeated pain assessment  ----------------------------------------------------------------------------------------------------------------------  Number of patients ≥ 75 years with documented pain treatment |  |

|  | **11.2**  During the course of treatment, a repeated pain assessment and, if applicable repeated/adapted measures of pain management should be performed. | **11.2.1 Process indicator (70 - 100%)**  Number of documented adapted pain treatment according to  ED specific guidelines for pain treatment  ----------------------------------------------------------------------------------------------------------------------  Number of all patients ≥ 75 years with documented pain calling out for treatment,  which already revised initial pain treatment in the ED |  |
| --- | --- | --- | --- |
|  |  | **11.2.2 Outcome indicator (50 - 100%)** |  |
|  |  | Number of documented pain reduction with repeated pain assessment  after adaption of pain therapy  ----------------------------------------------------------------------------------------------------------------------  Number of patients ≥ 75 years with documented adapted pain treatment |  |
| **12. Risk factor care needs** | **12.1**  Indwelling urinary catheters should only be inserted after documentation of the indication. They should be inserted according to current standards. | **12.1.1 Process indicator (90 - 100%)**  Number of documented insertions of permanent urinary catheters  ----------------------------------------------------------------------------------------------------------------------  Number of patients ≥ 75 years with documented indication for indwelling catheter | **12.3 Structural indicator**  A standard for the insertion of permanent urinary catheters according to current evidence is available. |
|  |  | **12.1.2 Process indicator (80 - 100%)** | **12.1.4 Structural indicator** |
|  |  | Number of documented insertions of permanent urinary catheters  according to current standard  ----------------------------------------------------------------------------------------------------------------------  Number of patients ≥ 75 years with insertion of a permanent urinary catheter during the ED stay | An appropriate evidence is provided that annually more than 80% of ED nursing staff have knowledge of the current standard for insertion of indwelling catheters. |
|  | **12.2**  In geriatric patients, assessment for pressure sores including identification of risk factors should be performed. | **12.2.1 Process indicator (90 - 100%)**  Number of documented assessments for pressure sores  ----------------------------------------------------------------------------------------------------------------------  Number of patients ≥ 75 years, excluding patients with the highest degree of urgency (according to a 5 level triage instrument) and only administratively registered patients. | **12.2.3 Structural indicator**  An appropriate evidence is provided that annually more than 80% of ED nursing staff have knowledge on the systematic assessment of pressure sore risk. |

|  |  | **12.2.2 Process indicator (90 - 100%)** |  |
| --- | --- | --- | --- |
|  |  | Number of documented systematic assessments of a pressure sore risk according to current recommendations of the expert's standard “Decubitus prevention in nursing” (P1)  ----------------------------------------------------------------------------------------------------------------------  Number of patients ≥ 75 years, excluding patients with the highest degree of urgency (according to a 5 level Triage instrument) and only administratively registered patients |  |
|  | **12.3**  In patients with pressure sores or at risk of pressure sores, an appropriate management according to current standards and guidelines should be initiated and documented. | **12.3.1 Process indicator (90 - 100%)**  Number of documented pressure releasing bedding plans including adequate time intervals, referring to the current recommendations of the expert's standard “Decubitus prevention in nursing” (P4)  ---------------------------------------------------------------------------------------------------------------------  Number of patients ≥ 75 years old, with pressure sores or risk of pressure sores, excluding patients with the highest degree of urgency (according to a 5 level triage instrument) and only administratively registered patients | **12.3.2 Structural indicator**  Sufficient aids for pressure distribution and pressure-relieving beddings are supplied to meet the needs of those who are identified as having pressure sores, or at risk of pressure sores. |
